# The natural analgesic conolidine targets the newly identified opioid scavenger ACKR3/CXCR7

**DOI:** 10.1038/s41392-021-00548-w

**Published:** 2021-06-02

**Authors:** Martyna Szpakowska, Ann M. Decker, Max Meyrath, Christie B. Palmer, Bruce E. Blough, Ojas A. Namjoshi, Andy Chevigné

**Affiliations:** 1 Immuno-Pharmacology and Interactomics, Department of Infection and Immunity, Luxembourg Institute of Health (LIH), Esch-sur-Alzette, Luxembourg; 2 Center for Drug Discovery, RTI International, Durham, NC USA; 3 Faculty of Science, Technology and Medicine, University of Luxembourg, Esch-sur-Alzette, Luxembourg

**Keywords:** Target identification, Drug screening

**Dear Editor**,

The atypical chemokine receptor ACKR3 has recently been reported to act as an opioid scavenger with unique negative regulatory properties towards different families of opioid peptides. Here, we show that conolidine, a natural analgesic alkaloid used in traditional Chinese medicine, targets ACKR3, thereby providing additional proof of a correlation between ACKR3 and pain modulation and opening alternative therapeutic avenues for the treatment of chronic pain.

In a recent study, we reported the identification and the characterization of a new atypical opioid receptor with unique negative regulatory properties towards opioid peptides.^1^ Our results showed that ACKR3/CXCR7, hitherto known as an atypical scavenger receptor for chemokines CXCL12 and CXCL11, is also a broad-spectrum scavenger for opioid peptides of the enkephalin, dynorphin, and nociceptin families, regulating their availability for classical opioid receptors. Gene expression analysis revealed that ACKR3 is highly expressed in several brain regions corresponding to important opioid activity centers. Additionally, its expression levels are often higher than those of classical opioid receptors, which further supports the physiological relevance of its observed in vitro opioid peptide scavenging capacity.

We demonstrated that, in contrast to classical opioid receptors, ACKR3 does not trigger classical G protein signaling and is not modulated by the classical prescription or analgesic opioids, such as morphine, fentanyl, or buprenorphine, or by nonselective opioid antagonists such as naloxone. Instead, we established that LIH383, an ACKR3-selective subnanomolar competitor peptide, prevents ACKR3’s negative regulatory function on opioid peptides in an ex vivo rat brain model and potentiates their activity towards classical opioid receptors. These results, together with a previous report showing that a small-molecule ACKR3 agonist CCX771 exhibits anxiolytic-like behavior in mice,^2^ support the concept of targeting ACKR3 as a unique way to modulate the opioid system, which could open new therapeutic avenues for opioid-related disorders.

Indeed, opioid drugs remain among the most widely prescribed analgesics to treat moderate to severe acute pain, but their use frequently leads to respiratory depression, nausea and constipation, as well as addiction and tolerance. These drawbacks have significantly reduced the treatment options of chronic and intractable pain and are largely responsible for the current opioid crisis.

There is an urgent need to find new means to modulate the opioid system, using drugs with alternative mechanisms of action and, crucially, improved safety profiles. Biased ligands, able to preferentially induce one signaling pathway over another, were regarded as a potential solution to this concern. Indeed, reports from β-arrestin-2 knockout mouse models proposed that side effects observed with opioids such as morphine were mainly linked to β-arrestin-dependent intracellular events. These findings fueled extensive efforts over the last years to find so-called “G protein-biased ligands”, i.e., agonists of opioid receptors that preferentially trigger G protein activation over arrestin recruitment, with the hope to improve pain management by fine-tuning opioid receptor-mediated signal transduction. Several ligands were discovered through these efforts, including the μ-opioid receptor (MOR) agonists PZM21 and TRV130 (Oliceridine, recently approved by the FDA), which show little β-arrestin recruitment or receptor internalization in in vitro models. Nevertheless, the improved therapeutic ratio of such molecules was recently proposed to stem from their low intrinsic efficacy rather than their biased profile,^3^ which further supports the importance of considering alternate strategies to modulate the opioid system.

Plants have been historically a source of analgesic alkaloids, although their pharmacological characterization is often limited. Among such natural analgesic molecules, conolidine, found in the bark of the tropical flowering shrub *Tabernaemontana divaricata*, also called pinwheel flower or crepe jasmine, has long been used in traditional Chinese, Ayurvedic and Thai medicines to treat fever and pain^4^ (Fig. 1a). Pharmacologists have only recently been able to confirm its medicinal and pharmacological properties thanks to its first asymmetric total synthesis.^5^ Conolidine is a rare C5-nor stemmadenine (Fig. 1b), which displays potent analgesia in in vivo models of tonic and persistent pain and reduces inflammatory pain relief. It was also suggested that conolidine-induced analgesia may lack complications usually associated with classical opioid drugs.^5^ Interestingly, conolidine was found to be present at micromolar levels in the brain after systemic injection^5^ but was not able to trigger direct activation of classical opioid receptors, notably MOR, and thus was not classified as an “opioid drug”. So far, the exact target and mechanism through which conolidine induces pain relief remain elusive. While conolidine was shown to bind to serotonin-3 ion channel, the norepinephrine transporter, N-type Cav2.2 calcium channels, as well as several GPCRs including the α2B adrenergic (ADRA2B), α2C adrenergic (ADRA2C), and histamine-2 (HRH2) receptors,^5^ the low affinity of these interactions suggests that they do not mediate the primary mechanism of action of conolidine.

**Fig. 1:**
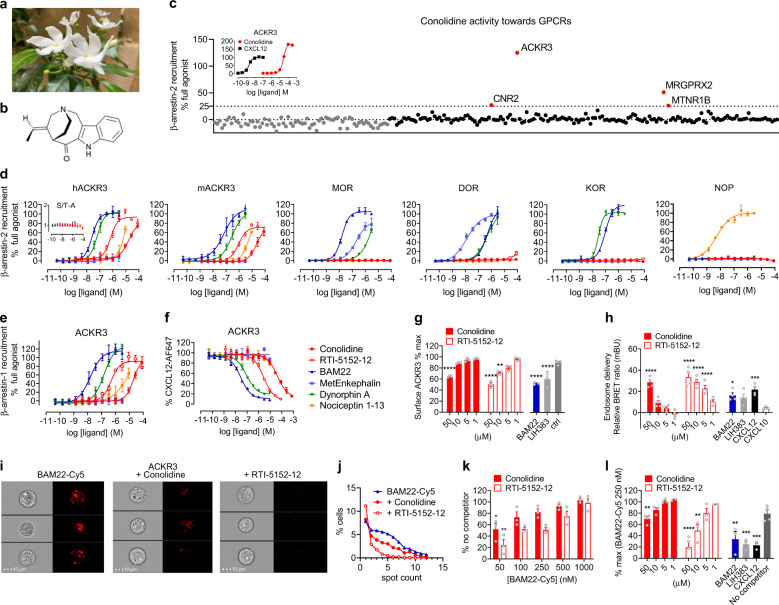
Conolidine acts as an agonist of the opioid scavenger ACKR3. **a** The pinwheel flower or Crepe Jasmine (*Tabernaemontana divaricata*). **b** Structure of conolidine with its C5-nor stemmadenine core. **c** Screening of conolidine (10 µM) towards the 74 GPCRs of orphanMAX panel (grey) and the 168 GPCRs of gpcrMAX panel (black) using a β-arrestin-2 recruitment assay based on β-galactosidase complementation (PathHunter, DiscoverX) in CHO-K1 cells. The full list of receptors and positive controls is available at www.discoverx.com. Inset. Potency and efficacy of CXCL12 and conolidine towards ACKR3 in a β-arrestin-2 recruitment assay (PathHunter). Conolidine triggered β-arrestin-2 recruitment to ACKR3 with a potency of 27 µM and efficacy of 185% compared to CXCL12. **d, e** Comparison of the potency and efficacy of conolidine and its analogue RTI-5152-12 with representative opioid peptides from the enkephalin (BAM22, Met-Enkephalin), dynorphin (Dynorphin A) and nociceptin (Nociceptin 1–13) family in activating human and mouse ACKR3 and the classical opioid receptors MOR, DOR, KOR, NOP using a β-arrestin-2 (**d**) or β-arrestin-1 (**e**) recruitment assay based on Nanoluciferase complementation (NanoBiT) in U87 cells. Inset. β-arrestin-2 recruitment to mutated ACKR3 lacking the C-terminal GRK phosphorylation sites (S/T-A) in response to conolidine, RTI-5152-12 and opioid peptides. Data are expressed as percentage of full agonist response: CXCL12 for ACKR3, BAM22 for MOR, Met-Enkephalin for DOR, Dynorphin A for KOR and Nociceptin 1–13 for NOP or as fold baseline for inset. Conolidine triggered β-arrestin-2 recruitment to human and mouse ACKR3 with potency of 16 µM and 22 µM, respectively, and β-arrestin-1 recruitment to human ACKR3 with potency of 19 µM. **f** Binding competition of conolidine and its analogue RTI-5152-12 and representative opioid peptides with Alexa Fluor 647-labeled CXCL12 (5 nM) on U87-ACKR3 cells determined by flow cytometry. The experiment was performed on ice to prevent ligand-induced internalization. **g** ACKR3 internalization in response to conolidine and its analogue RTI-5152-12 in comparison to BAM22, LIH383 and control peptide (1 µM) used as positive and negative controls. Following a brief acidic wash to remove receptor-bound ligands, the presence of ACKR3 at the cell surface was monitored by flow cytometry using an anti-ACKR3 mAb (clone 11G8). **h** ACKR3 delivery to the early endosomes in response to conolidine and RTI-5152-12, CXCL12 (1 µM) or peptides BAM22 and LIH383 (1 µM) monitored by NanoBRET-based assay in U87 cells using ACKR3-Nanoluciferase as donor and FYVE domain of endofin, interacting with phosphatidylinositol 3-phosphate (PI3P) in early endosomes, fused to the mNeonGreen fluorescent protein, as acceptor. Chemokine CXCL10 was used as negative control. **i** ACKR3-mediated uptake of Cy5-labeled BAM22 (250 nM) in competition with conolidine and RTI-5152-12 (50 µM) visualized in U87-ACKR3 cells by imaging flow cytometry. Three representative cells per condition are shown out of 5000 single, in focus, living cells recorded. Scale bar: 10 µm. **j** Percentage of cells with a given number of distinguishable vesicle-like structures (spots) representative of three independent experiments. **k** ACKR3-mediated uptake of Cy5-labeled BAM22 (50 nM–1 µM) in competition with conolidine (50 µM) or RTI-5152-12 (10 µM) visualized in U87-ACKR3 cells by imaging flow cytometry. **l** Uptake competition between Cy5-labeled BAM22 (250 nM) and varying concentrations of conolidine and RTI-5152-12 (50, 10, 5, 1 µM) or CXCL12, BAM22 and LIH383 (1 µM) used as positive controls in U87-ACKR3 cells. Data are presented as mean ± S.E.M. of three (four for h) independent experiments (*n* = 3 or *n* = 4). *p < 0.05, **p < 0.01, ***p < 0.001, ****p < 0.0001 by one-way ANOVA with Bonferroni’s post hoc test (**g, h**, **l**) or repeated measures one-way ANOVA with Dunnet’s post hoc test (**k**). Source data are provided as a Source Data file

In a recent joint effort to identify the target of conolidine among the GPCR family, we undertook a large scale screening program using a β-arrestin recruitment assay based on β-galactosidase complementation (PathHunter, DiscoverX). Over 240 receptors were tested for their ability to be activated or inhibited by conolidine (10 µM) (Fig. 1c). These included 168 GPCRs from the gpcrMAX panel, covering over 60 distinct receptor families such as adrenergic, dopamine, P2Y, or serotonin and 74 GPCRs from the orphanMAX panel. The screening pinpointed ACKR3 as the most responsive receptor modulated by conolidine. No activity was detected towards ADRA2B, ADRA2BC, and HRH2, whereas the Mast cells G protein-coupled receptor-X2 (MRGPRX2), the cannabinoid receptor 2 (CNR2) and the melatonin receptor 1B (MTNR1B) were only partially activated by conolidine. Further confirmation and characterization efforts using PathHunter and NanoBiT technologies demonstrated that conolidine acts as a full agonist of the newly identified opioid peptide scavenger ACKR3 (Fig. 1c, d and e). Although less potent than natural endogenous opioid peptides, conolidine was able to bind both human and mouse ACKR3 acting as a full agonist triggering receptor phosphorylation and subsequent recruitment of β-arrestin-1 and -2 with micromolar potencies (Fig. 1d and e), while showing no activity towards classical opioid receptors (MOR, DOR, KOR, and NOP). Conolidine also competed with the binding of fluorescently labeled chemokine CXCL12 (Fig. 1f) and the opioid peptide BAM22 (Fig. 1i, k and l) to the receptor, induced ACKR3 internalization (Fig. 1g) and its subsequent delivery to the endosomes (Fig. 1h). Interestingly, systematic chemical modifications of conolidine resulted in a novel compound (RTI-5152-12) with 15-fold improved potency towards ACKR3 (Fig. 1d, e and f) and both the natural conolidine and its analogue were able to restrain the uptake of the endogenous opioid peptide BAM22 by ACKR3 in a concentration-dependent manner (Fig. 1i, j, k and l).

Therefore, in analogy to LIH383,^1^ conolidine may inhibit the scavenging functions of ACKR3, consequently increasing the availability of pain relief-inducing endogenous opioid peptides for the classical opioid receptors. The present study demonstrates that conolidine, an alkaloid with in vivo analgesic properties, is a modulator of ACKR3. These results further support the proposed link between ACKR3 and the opioid system and strengthen our initial findings that ACKR3 is an atypical opioid receptor (AOR) and that its modulation may have a direct impact on nociception. Although polypharmacology of conolidine cannot be excluded, this compound may qualify as an opioid drug, which targets atypical over classical opioid receptors. In this context, synthetic conolidine analogues and other alkaloids structurally similar to conolidine, such as apparicine, pericine, or stemmadenine, should also be tested for their ability to modulate ACKR3. Overall, the discovery of the potential mode of action of conolidine and its activity on ACKR3 is a significant step forward towards a more exhaustive understanding of its role in pain regulation, bearing great potential for novel drug development against chronic pain.

## Supplementary information

Supplementary Materials

## Data Availability

The datasets used to support the findings of this study are available from the corresponding authors upon reasonable request.
