# Supplementary material for: The natural analgesic conolidine targets the newly identified opioid scavenger ACKR3/CXCR7
Source: Signal Transduct Target Ther. 2021 Jun 2;6:209. doi: 10.1038/s41392-021-00548-w (PMC8169647; doi:10.1038/s41392-021-00548-w)
Supplement: Supplementary file 1 — Supplementary Materials [file 41392_2021_548_MOESM1_ESM.docx]

**Supplementary Materials for**

**The natural analgesic conolidine targets the newly identified opioid scavenger ACKR3/CXCR7**

Szpakowska *et al.*

This file includes:

- Material and methods
- Supplementary references

***Peptides and chemokines***

Non-labeled chemokines CXCL12 and CXCL10 were purchased from PeproTech. Alexa Fluor 647-labeled CXCL12 (CXCL12-AF647) was purchased from Almac. The opioid peptides were acquired from Phoenix Pharmaceuticals. Peptide LIH383 was synthesized by JPT. BAM22 labeled with Cy5 was generated using an Amersham QuickStain Cy5 kit for proteins according to manufacturer’s protocol.

***Cell culture***

U87 cells derived from human glioblastoma were obtained through the NIH AIDS Reagent Program and grown in Dulbecco’s modified Eagle medium (DMEM) supplemented with 15 % fetal bovine serum and penicillin/streptomycin (100 Units per ml and 100 µg per ml). U87-ACKR3 cells^1^ were maintained under puromycin selective pressure (1 µg per ml). Cells were regularly tested for mycoplasma contamination.

***β-galactosidase complementation-based β-arrestin recruitment assay***

Conolidine-induced β-arrestin-2 recruitment towards the 74 GPCRs of orphanMAX panel and the 168 GPCRs of the gpcrMAX panel was monitored by β-galactosidase complementation assay (PathHunter, DiscoverX). In brief, CHO-K1 cells stably expressing β-arrestin-2 fused to the enzyme acceptor of β-galactosidase and the GPCR fused to the β-galactosidase ProLink donor were seeded 24 hours before the experiment in 384-well plate. Conolidine (10 μM) was then added and after 90-minute incubation at 37 °C, luminescent signal was generated through addition of β-galactosidase substrate (PathHunter detection reagent). After 1-hour incubation at room temperature, chemiluminescent signal was measured on a PerkinElmer EnvisionTM plate reader. The full list of receptors and positive controls is available at [www.discoverx.com](http://www.discoverx.com).

***Nanoluciferase complementation-based β-arrestin recruitment assay***

Ligand-induced β-arrestin recruitment to chemokine and opioid receptors was monitored by NanoLuc complementation assay (NanoBiT, Promega)^2,3^. In brief, 1.2 × 10^6^ U87 cells were plated in 10-cm culture dishes and 48 hours later co-transfected with pNBe vectors encoding GPCRs C-terminally tagged with SmBiT and human β-arrestin-1 (arrestin-2) or β-arrestin-2 (arrestin-3) N-terminally fused to LgBiT. 48 hours after transfection cells were harvested, incubated 25 minutes at 37 °C with Nano-Glo Live Cell substrate diluted 200-fold and distributed into white 96-well plates (5 × 10^4^ cells per well). Ligand-induced β-arrestin recruitment to GPCRs was evaluated by measuring luminescence signal for 20 minutes with a Mithras LB940 luminometer (Berthold Technologies, running on MicroWin 2010 5.19 software (Mikrotek Laborsysteme).

***Chemokine and opioid peptide binding competition assays***

U87-ACKR3 cells were distributed into 96-well plates (1.5 × 10^5^ cells per well) and incubated with a mixture of CXCL12-AF647 (5 nM) and conolidine, RTI-5152-12 or opioid peptides at indicated concentrations for 90 minutes on ice. Cells were then washed twice with FACS buffer (PBS, 1 % BSA, 0.1 % NaN_3_) at 4 °C. Dead cells were excluded using Zombie Green viability dye (BioLegend, dilution 1:2000). ACKR3-negative U87 cells were used to evaluate non-specific binding of CXCL12-AF647. 0 % receptor binding of CXCL12-AF647 was defined as the signal obtained after addition of 1 µM of unlabeled CXCL12. The signal obtained for CXCL12-AF647 in the absence of unlabeled chemokines was used to define 100 % binding. Ligand binding was quantified by mean fluorescence intensity on a BD FACS Fortessa cytometer (BD Biosciences) using FACS Diva 8.01 (BD Biosciences).

***Ligand-induced changes in receptor cell surface levels***

For the determination of ACKR3 surface expression levels by flow cytometry, U87-ACKR3 cells were stimulated with conolidine, RTI-5152-12 or positive and negative controls for 60 minutes at 37 °C. The remaining surface-bound ligands were then removed by a brief low-pH wash (150 mM NaCl, 50 mM glycine, pH 3) and twice with FACS buffer (PBS, 1 % BSA, 0.1 % NaN_3_). Cell surface levels of ACKR3 were then measured by flow cytometry using a saturating concentration (12.5 µg per ml, dilution 1:40) of receptor-specific mAb (clone 11G8 R&D Systems, cat# MAB42273) and a secondary phycoerythrin–conjugated F(ab’)_2_ fragment anti-mouse IgG (dilution 1:300, Jackson ImmunoResearch cat# 115-116-071). Dead cells were excluded using Zombie NIR viability dye (BioLegend, dilution 1:2000). Mean fluorescence intensity was quantified on a BD FACS Fortessa cytometer (BD Biosciences) using FACS Diva 8.01 (BD Biosciences).

***Ligand-induced receptor delivery to endosomes***

Ligand-induced receptor delivery to endosomes was monitored by NanoBRET. In brief, 1.2 × 10^6^ U87 cells were seeded in 10-cm dishes and 24 hours later co-transfected with plasmids encoding ACKR3 C-terminally tagged with Nanoluciferase and FYVE domain of endofin, interacting with phosphatidylinositol 3-phosphate (PI3P) in early endosomes^4,5^, C-terminally tagged with mNeonGreen. After 24 hours, cells were detached and distributed into black 96-well plates (1 × 10^5^ cells per well) and treated with indicated concentrations of ligands. After 150-minute incubation at 37 °C, coelenterazine H (10 µM) was added and donor emission (460 nm) and acceptor emission (535 nm) were immediately measured on a GloMax plate reader (Promega).

***Visualization of fluorescently labeled opioid peptide uptake***

U87-ACKR3 cells were distributed into 96-well plates (3 × 10^5^ cells per well in Opti-MEM). After 15-minute incubation at 37 °C with conolidine, RTI-5152-12, control peptides at indicated concentrations or Opti-MEM only, BAM22-Cy5 (250 nM, unless indicated differently) was added, incubated for 40 minutes at 37 °C and washed twice with FACS buffer (PBS, 1 % BSA, 0.1 % NaN_3_). Dead cells were excluded using Zombie Green viability dye (BioLegend, dilution 1:2000). Images of 5 × 10^3^ in-focus living single cells were acquired with an ImageStream MKII imaging flow cytometer (Amnis, running on the INSPIRE Mark II software (EMD Millipore)) using 40x magnification. Samples were analyzed using Ideas6.2 software. The number of spots per cell was determined using a mask-based software wizard.

***Data and statistical analysis***

Data are reported as mean ± S.E.M.. Sample size was chosen according to the standards of the field (at least three independent biological replicates for each condition). Concentration–response curves were fitted to the four-parameter Hill equation using an iterative, least-squares method (GraphPad Prism version 8.0.1). For the comparison of three or more sets of unpaired measurements, ordinary one-way ANOVA was applied with Bonferroni’s post hoc test. For analysis of matched groups, a repeated measures one-way ANOVA was applied with Dunnet’s post hoc test comparing every mean to the mean of untreated control. ANOVA and post hoc analyses were performed with GraphPad Prism 8.0.1. P-values are indicated as follows: *p < 0.05, **p < 0.01, ***p < 0.001, ****p < 0.0001.

***Supplementary references***

1 Szpakowska, M. *et al.* Different contributions of chemokine N-terminal features attest to a different ligand binding mode and a bias towards activation of ACKR3/CXCR7 compared with CXCR4 and CXCR3. *Br J Pharmacol* **175**, 1419-1438, doi:10.1111/bph.14132 (2018).

2 Dixon, A. S. *et al.* NanoLuc Complementation Reporter Optimized for Accurate Measurement of Protein Interactions in Cells. *ACS Chem Biol* **11**, 400-408, doi:10.1021/acschembio.5b00753 (2016).

3 Szpakowska, M. *et al.* Mutational analysis of the extracellular disulphide bridges of the atypical chemokine receptor ACKR3/CXCR7 uncovers multiple binding and activation modes for its chemokine and endogenous non-chemokine agonists. *Biochem Pharmacol* **153**, 299-309, doi:10.1016/j.bcp.2018.03.007 (2018).

4 Schink, K. O., Raiborg, C. & Stenmark, H. Phosphatidylinositol 3-phosphate, a lipid that regulates membrane dynamics, protein sorting and cell signalling. *Bioessays* **35**, 900-912, doi:10.1002/bies.201300064 (2013).

5 Namkung, Y. *et al.* Monitoring G protein-coupled receptor and beta-arrestin trafficking in live cells using enhanced bystander BRET. *Nat Commun* **7**, 12178, doi:10.1038/ncomms12178 (2016).
